# Synergistic Effect of Polydeoxyribonucleotides with Low-Level Lasers on the Regeneration of Crush-Injured Facial Nerves

**DOI:** 10.3390/jcm14051678

**Published:** 2025-03-01

**Authors:** Kyung Hoon Sun, Cheol Hee Choi, Chul Ho Jang

**Affiliations:** 1 Department of Emergency Medicine, College of Medicine, Chosun University, Gwangju 61452, Republic of Korea; skhkorea@chosun.ac.kr; 2 Department of Pharmacology, College of Medicine, Chosun University, Gwangju 61452, Republic of Korea; chchoi@chosun.ac.kr; 3 Department of Otolaryngology, Gwangju Veterans Hospital, Gwangju 62284, Republic of Korea; 4 Department of Otolaryngology, Chonnam National University Medical School, Gwangju 61469, Republic of Korea

**Keywords:** facial nerve paralysis, PDRN, low-level laser therapy, regeneration

## Abstract

**Background/Objectives**: The regeneration of the facial nerve using low-level laser therapy (LLLT) has been infrequently reported. Polydeoxyribonucleotides (PDRNs), a blend of short deoxyribonucleotide polymers known for their non-toxic and non-allergic properties, are recognized as a stimulator of cell growth that enhances cell proliferation and supports wound healing. This study investigates the synergistic effect of the topical sustained release of PDRN/F-127 and LLLT on facial nerve regeneration following crush injury-induced paralysis in rats. **Methods**: The main trunk of the facial nerve was compressed for 1 min using a hemostat. Animals were divided into five groups: a control group (n = 4), group I (Pluronic F-127 only, n = 4), group II (Pluronic F-127/PDRN, n = 4), group III (Pluronic F-127 + LLLT, n = 4), and group IV (Pluronic F-127/PDRN + LLLT, n = 4). We measured the recovery of vibrissa fibrillation, action potential, and facial nerve blood flow (FNBF). **Results**: Group IV exhibited a comparatively faster development of vibrissa fibrillation over time than the other groups. After the intervention, significant differences in vibrissa fibrillation values were observed at all time points (*p* = 0.0028) according to the repeated one-way ANOVA. Regarding the threshold of action potential, all five groups revealed a significant difference (one-way ANOVA, *p* < 0.0001; multiple comparisons via Tukey’s test). Among the groups, group IV showed a significantly reduced threshold of action potential compared to the other groups. Group IV showed the most notable recovery in FNBF compared to the other groups. One-way ANOVA showed a significant difference (*p* < 0.0001; multiple comparisons by Dunnett’s test). **Conclusions**: These findings suggest that PDRN and LLLT may work together synergistically to enhance peripheral nerve regeneration. Future studies should investigate the underlying molecular mechanisms and evaluate the potential clinical applications of this combined treatment strategy.

## 1. Introduction

Facial nerve palsy is characterized by paralysis affecting all areas innervated by the facial nerve, leading to diminished facial expression. Trauma is responsible for 10–23% of the causes of facial nerve paralysis [1]. Notably, this condition significantly affects patients’ quality of life, with facial trauma being the leading cause of traumatic facial nerve paralysis. Unlike the central nerves, the peripheral nerves can regenerate functionally after injury by extending towards the damaged region [2]. Damage to peripheral facial nerves alters the expression of different neuroactive substances that impact the growth, survival, regeneration, and damage of nerve cells. Damage to the peripheral facial nerve directly impacts the peripheral nerves and affects the central nervous system (CNS) through a variety of factors. However, it is unclear which substances are responsible for these CNS changes [2]. Traumatic facial nerve paralysis results in regeneration, although it may occur slowly. For severe or complete nerve damage, surgery is the primary treatment option. Innovated surgical methods, including autografts and allografts, alongside advancements in material science and engineering, have been developed to repair injured nerves. Additionally, non-surgical approaches have emerged to facilitate nerve regeneration, whether as a supplementary therapy post-surgery or as the main treatment for axonotmesis [2,3,4]. As such, rehabilitation programs should be prioritized for the treatment of nerve injuries to maintain adequate facial muscle tone and deter further facial muscle atrophy.

Photobiomodulation therapy (PBMT) employing low-level laser therapy (LLLT) has been shown to enhance peripheral nerve regeneration post-trauma [3]. The wavelength of PBMT application protocols in nerve lesions has been found to vary widely, ranging from 632.8 to 904 nm, along with diverse energy levels and application durations, despite the similar lesion types investigated in each study [4,5]. While most research has concentrated on sciatic nerve regeneration [6,7], reports on facial nerve regeneration using PBMT are scarce. To date, only three papers have been published on this topic [8,9,10]. Among these studies, Yuca et al. [10] noted that PBMT improved the healing process when compared to a control group, although the outcome was not statistically significant.

Pluronic F127 (F127) is an FDA-approved hydrogel composed of polyoxyethylene–polyoxypropylene–polyoxyethylene (PEO-PPO-PEO), combining hydrophilic and lipophilic properties. Spherical microgels are produced when the hydrophobic PPO block dehydrates and cross-links with the hydrophilic PEO block at physiological temperatures (about 37 °C) [11,12,13,14]. The thermo-reversible gel F127 has been used in a variety of industries because of its high thermos-sensitivity, biodegradability, and biocompatibility qualities [15]. As reported previously, Pluronic F127 is a temperature-sensitive, injectable, biodegradable, nonionic, non-toxic, and biocompatible polymer [16]. This polymer’s characteristics allow it to be used as a flexible drug carrier. By enhancing the bioavailability and thermostability of medications, F127 increases its therapeutic efficacy [13]. Furthermore, it has the ability to encourage collagen synthesis and cellular attachment, which supports angiogenesis and tissue regeneration [15]. The encapsulation of mesenchymal stem cells using F127 has proven effective in promoting the regeneration of tissues with low vascularity, including adipose, cartilage, tendons, bone, and epithelial tissues [17,18].

A mixture of short deoxyribonucleotide polymers, polydeoxyribonucleotides (PDRNs), are known to be non-toxic agonists with no antigenic qualities. They also function as a powerful growth-stimulating agent that promotes cell proliferation and wound healing [19]. PDRNs can be derived from a placental extract or from trout or salmon sperm [20,21].

It is noted for improving angiogenesis during wound healing [20,22,23,24]. However, studies examining the effect of PDRNs on neural regeneration are limited. We postulated that topical PDRN, either by itself or in conjunction with the LLLT, would be more synergistic in addressing nerve regeneration processes.

This study aimed to examine the potential synergistic effects of PDRN in conjunction with PBMT using a low-level laser on crush-injured facial nerve paralysis in a rat model.

## 2. Materials and Methods

### 2.1. Induction of Crush Injury-Induced Facial Nerve Paralysis and Treatment

Twenty adult Sprague Dawley rats (6–8 weeks old, weighing 250–300 g) were used in the experiment, which was approved by Chosun University (CIACUC2024-SOOO8, 15 February 2024). Each rat was housed in a separate cage and provided with feed and water. They were allowed to adapt to the environment without stress for a week before surgery. A postauricular incision was made to access the main trunk of the facial nerve after the left auricle was shaved. The left side was chosen for easier setup during action potential threshold testing. The main trunk of the facial nerve was revealed by dissecting the subcutaneous layer under surgical microscopy. The main trunk was then crushed for 1 min using a hemostat, ensuring damage to all nerve fibers while preserving the axonal sheath. Each rat was housed individually with food and water ad libitum. To minimize stress, the animals were given a week to acclimate to their environment before the experiment began.

### 2.2. Animal Group and Each Treatment

Animals were allocated into five groups: control (n = 4), group I (Pluronic F-127 only, n = 4), group II (Pluronic F-127 combined with PDRN, n = 4), group III (Pluronic F-127 plus LLLT, n = 4), and group IV (Pluronic F-127/PDRN with LLLT, n = 4).

**Figure 1:**
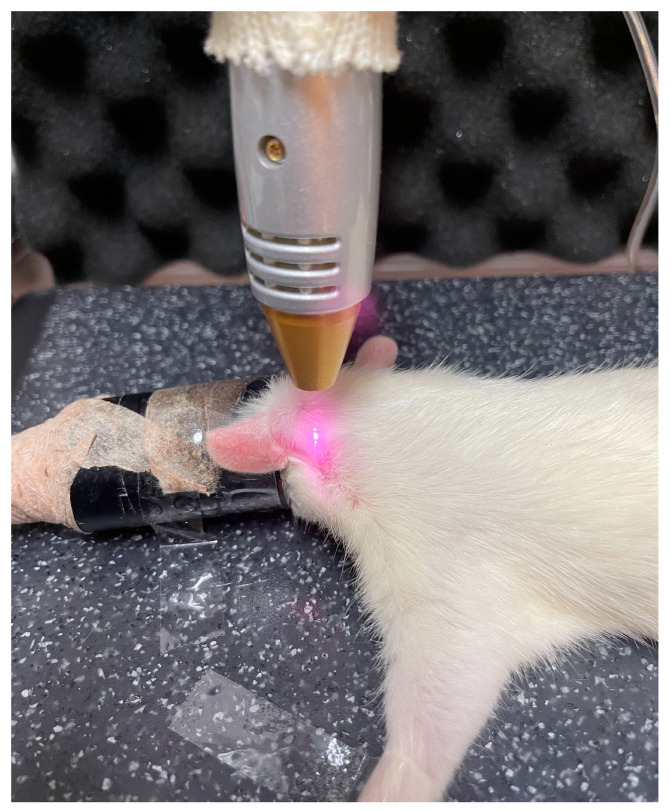
The LLLT was performed 40 s/day for 3 weeks post-injury.

To ensure the sustained release of PDRN to the crushed injured facial nerve, PDRN (8 mg/kg) was combined with 20% Pluronic F-127 in PBS. This PDRN/Pluronic F-127 mixture was applied topically immediately after inducing the facial nerve crush injury. The LLLT (ARAPA 890, SAEIM Medical Co., Gyeonggi-do, Republic of Korea) treatment was administered for 40 s (wave 830 nm; power 230 mw, continuous) daily over 3 weeks post-injury (Figure 1).

### 2.3. Evaluation of Vibrissa Movement Recovery Using Slow Motion Video Analysis Software

The rat’s head remained exposed while its body was secured in a modified plastic bottle. We used an iPhone (Apple Inc., Los Altos, CA, USA) to record video of vibrissa movement on the left side for all five groups at 1, 2, and 3 weeks after tactile stimulation with a brush aimed at addressing facial nerve paralysis from a crush injury. The behavioral observation research interactive program (BORIS), developed to analyze animal behavior, was employed to track vibrissa fibrillation in slow motion. The software, created by Oliver Friard and Marco Gamba at the University of Turin’s Department of Life Sciences and Systems Biology, is publicly accessible for research purposes. The frequency of vibrissa movement was compared between the left side and the right normal side.

### 2.4. Electrically Induced Action Potential Measurement

Four weeks after surgery, the facial nerves were reexplored while the rat was under general anesthesia with isoflurane inhalation. A monopolar tungsten probe stimulated the distal end of the crush site, determining the action potential threshold as described previously [25]. These two-needle electrodes were positioned percutaneously at the midpoint of the left orbicularis oculi and orbicularis oris muscles. One grounding electrode served to capture electrically evoked muscle action potential (MAP) signals from the superficial muscle layer near the skin. Using a monopolar stimulating electrode (Xomed-Treace, Jacksonville, FL, USA) coupled to a pulse generator, the main trunk of the facial nerve was electrically stimulated by a nerve stimulator (A-320D; World Precision, Sarasota, FL, USA) with a rectangular current pulse for 0.05 ms.

A micromanipulator was used to position and direct the monopolar stimulating probe in relation to the facial nerve. MAP signals were recorded during maximal nerve stimulation. Data collection was automated using a Samsung monitor and the lab chart system (PowerLab; AD Instrument, Castle Hill, Australia). The data were then analyzed with the AD Instruments Scope program (LabChart 8.1 system (PowerLab; AD Instrument, Castle Hill, Australia)), focusing on the peak amplitude of the action potential waveform to evaluate recovery following facial nerve injury.

### 2.5. Using a Laser Doppler Blood Flowmeter to Quantify Nerve Blood Flow

At 4 weeks post-surgery, two rats from each group were anesthetized using an intraperitoneal injection of tiletamine-zolazepam (Zoletil, Virbac, Carros, France) and xylazine hydrochloride. Combining Zoletil with xylazine hydrochloride presents a more practical approach for measuring facial nerve blood flow (FNBF). The femoral artery was located, and the primary trunk of the face nerve was carefully re-exposed. As stated earlier [26], FNBF in the specified area was measured using a laser Doppler blood flowmeter. A pressure transducer was attached to the cannulated femoral artery, which is typically used for systemic blood pressure (SBP) (AD Instruments, Castle Hill, Australia). To avoid nerve compression, a 1.0 mm needle probe was meticulously placed on the main trunk of the FN and linked to a laser Doppler blood flowmeter (moorLAB, Moor Instruments, Axminster, Devon, UK). Data sampling and analysis of the FNBF output and SBP were performed every 20 s using a laptop (Samsung, Suwon, Republic of Korea) and a data acquisition tool (PowerLab, AD Instruments, Castle Hill, Australia). FNBF was recorded for 30 min.

### 2.6. Statistical Analysis

GraphPad Prism 8.0 was used for all statistical analyses. A one-way ANOVA compared the five groups with a *p*-value considered statistically significant if it was below 0.05.

## 3. Results

### 3.1. Recovery of Vibrissa Fibrillation

**Figure 2:**
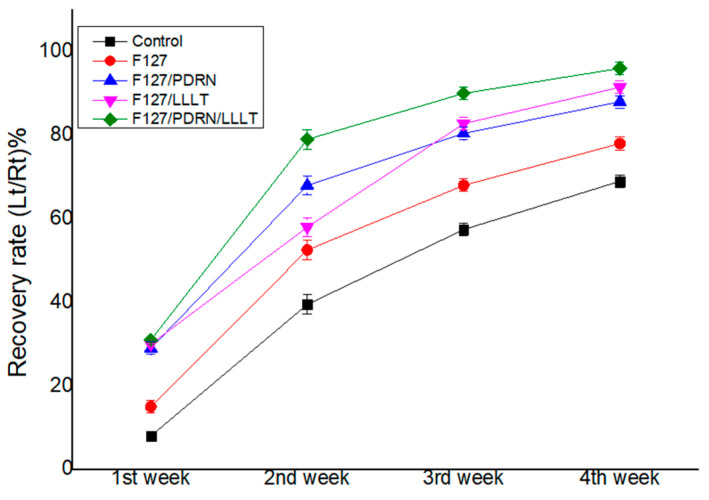
Over time, vibrissa fibrillation appeared in group IV relatively faster than in the other groups. After the intervention, repeated one-way ANOVA showed a significant group difference (*p* = 0.0028) in the vibrissa fibrillation levels at all time intervals. There was a significant difference between the postoperative weeks (*p* < 0.0001).

Figure 2 illustrates the sequential recovery of vibrissa fibrillation for each group at four postoperative time points. Group IV exhibited a comparatively faster development of vibrissa fibrillation over time than the other groups. After the intervention, significant differences in vibrissa fibrillation values were observed at all time points (*p* = 0.0028) according to the repeated one-way ANOVA. Additionally, a significant difference was noted between the postoperative weeks, with *p* < 0.0001 (Figure 2).

### 3.2. Recovery of Facial Muscle Action Potential

**Figure 3:**
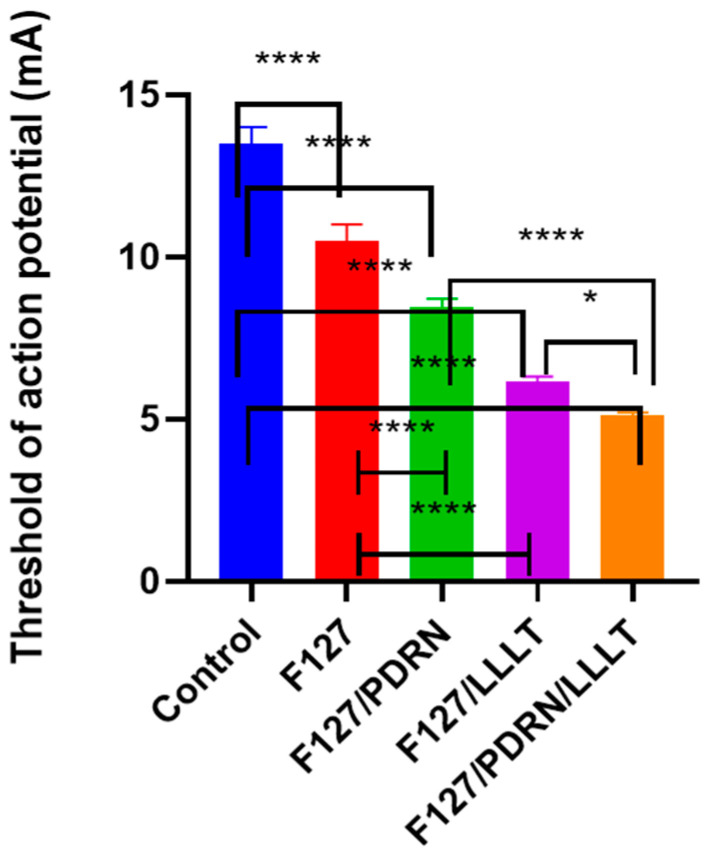
Group IV exhibited a much lower threshold than the other groups. Each of the four groups showed a significant difference (one-way ANOVA, *p* < 0.0001; multiple comparisons by Tukey’s test, control vs. group I, *p* < 0.0001; control vs. group II, *p* < 0.0001; control vs. group III, *p* < 0.0001; control vs. group IV, *p* < 0.0001; group I vs. group II, *p* = 0.0002; group I vs. group III, *p* < 0.0001; group I vs. group IV, *p*< 0.0001; group II vs. group III, *p* < 0.0001; group II vs. Group IV, *p* < 0.0001; group III vs. group IV, *p* = 0.0234. * means *p* < 0.05, **** means *p* < 0.0001.

Four weeks after the crush injury, as depicted in Figure 3, all five groups revealed a significant difference (one-way ANOVA, *p* < 0.0001; multiple comparisons via Tukey’s test, control vs. group I, *p* < 0.0001; control vs. group II, *p* < 0.0001; control vs. group III, *p* < 0.0001; control vs. group IV, *p* < 0.0001; group I vs. group II, *p* = 0.0002; group I vs. group III, *p* < 0.0001; group I vs. group IV, *p* < 0.0001; group II vs. group III, *p* < 0.0001; group II vs. group IV, *p* < 0.0001; group III vs. group IV, *p* = 0.0234). Among the groups, group IV showed a significantly reduced threshold compared to the other groups.

### 3.3. Recovery of Facial Nerve Blood Flow

**Figure 4:**
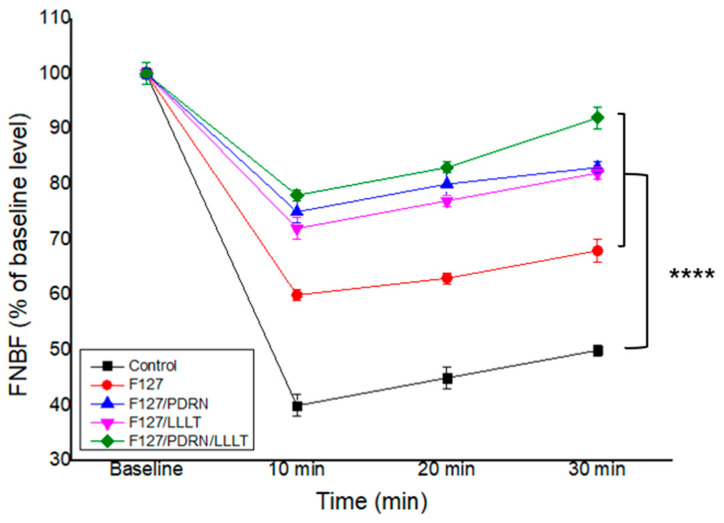
The FNBF of the other groups was significantly better than that of the control group. Group IV’s FNBF recovery was noticeably better than those of the other groups. One-way ANOVA showed significant difference (**** *p* < 0.0001; multiple comparisons by Dunnett’s test, control vs. group I, *p* < 0.0001; control vs. group II, *p* < 0.0001; control vs. group III, *p* < 0.0001; control vs. group IV, *p* < 0.0001).

Compared with the sham control group, other groups demonstrated a significant recovery in FNBF. Group IV showed the most notable recovery in FNBF compared to the other groups (Figure 4). One-way ANOVA showed a significant difference (*p* < 0.0001; multiple comparisons by Dunnett’s test, control vs. group I, *p* < 0.0001; control vs. group II, *p* < 0.0001; control vs. group III, *p* < 0.0001; control vs. group IV, *p* < 0.0001). This remarkable recovery in group IV is attributed to the synergistic effects of PDRN and LLLT.

## 4. Discussion

This study examined the combined effects of PDRN and LLLT on facial nerve regeneration after crush injury in an animal model. Our findings revealed that the integration of PDRN and LLLT (Group IV) significantly improved nerve recovery, which was indicated by the quicker development of vibrissa fibrillation, the enhanced recovery of the action potential threshold, and increased blood flow to the nerves compared to the other groups. Vibrissa fibrillation, an essential marker of facial nerve function [27], appeared more swiftly in Group IV than in the other groups, implying that the joint treatment accelerates neuromuscular recovery.

Statistically significant differences recorded at all time points and across postoperative weeks confirmed the synergistic effects of PDRN and LLLT on nerve repair processes. Additionally, Group IV consistently showed superior performance in multiple comparisons via Tukey’s test, highlighting the exceptional regenerative benefits of this combination therapy. The recovery of action potential thresholds was also significantly better in Group IV, suggesting that PDRN and LLLT work together to support the effective restoration of nerve excitability and conduction, which is a critical marker for successful nerve regeneration, as lower thresholds reflect improved neural transmission through the injured nerve segment.

The increase in nerve blood flow in Group IV, measured through laser Doppler blood flowmetry, further emphasized the role of PDRN and LLLT in enhancing peripheral nerve regeneration. Improved blood flow is crucial for providing the oxygen and nutrients necessary for nerve repair. Previous research has demonstrated that both PDRN and LLLT enhance angiogenesis and microcirculation in damaged tissues when used individually [28,29]. The combination of these two therapies appears to amplify this effect, contributing to the enhanced nerve regeneration seen in our study.

The anti-apoptotic signals triggered by PDRN lead to increased levels of cyclic adenosine monophosphate (cAMP) and an elevated Bax/Bcl-2 ratio. Multiple studies indicate that PDRN exhibits anti-inflammatory effects across various cell lines [30,31,32,33]. Specifically, in human chondrosarcoma cells stimulated by IL-1 (10 ng/mL) and RAW 264.7 cells activated by zoledronic acid (ZA, 10 M) and LPS (0.1 g/mL), PDRN demonstrated anti-inflammatory properties [33].

According to these findings, pro-inflammatory cytokines like IL-1 and IL-6 are downregulated by OK-PDRN, which reduces swelling [30]. Moreover, at a concentration of 100 g/mL, PDRN was found to enhance the production of the anti-inflammatory cytokine IL-10 in RAW cells while simultaneously suppressing nitric oxide (NO) synthesis and the production of pro-inflammatory cytokines like IL-12 and TNF-α [31].

LLLT is an effective treatment for peripheral nerve regeneration. Via stimulation, laser therapy promotes collagen formation, cell proliferation, improved blood circulation, and nerve regeneration. LLLT is also recognized for its analgesic, anti-inflammatory, and anti-edematous properties [34]. Rochkind et al. [35] noted that LLLT improves peripheral nerve function, resulting in significant functional recovery.

In this study, we incorporated PDRN with F127. The thermo-sensitive, biodegradable, and biocompatible Pluronic F127 is a thermo-reversible gel widely used across different fields [15]. These properties enable it to serve as a versatile drug carrier. Pluronic F127 enhances both drug bioavailability and thermostability, thereby elevating their therapeutic effectiveness [36]. Additionally, it promotes the synthesis of collagen and cellular adhesion, aiding angiogenesis and tissue regeneration. Due to the efficacy of Pluronic F127, mesenchymal stem cells (MSCs) have been encapsulated to facilitate the regeneration of low-vascularity tissues such as adipose tissue, cartilage, tendons, and bone [15]. In this research, the effects of angiogenesis and tissue F-127 on angiogenesis and tissue regeneration, along with the recovery of vibrissa fibrillation and FNBF, were significantly greater than those in the sham control group.

The hypotheses concerning the synergy between PDRN and LLLT suggest mechanisms that may include the modulation of inflammation, fostering angiogenesis, and enhancing cellular proliferation and collagen synthesis. PDRN, a DNA-based therapeutic agent, is known to activate adenosine A2A receptors, leading to tissue repair and anti-inflammatory effects.

LLLT, on the other hand, is established as a promoter of cellular proliferation and angiosome modulation through the photobiomodulation of mitochondrial functions. Together, these approaches could yield complementary advantages that expedite nerve regeneration.

Our findings interestingly revealed the individual advantages of both PDRN and LLLT in promoting nerve regeneration. Nonetheless, the notably superior results in Group IV imply that their combination is more effective than each therapy on its own. This holds significant implications for developing integrated therapeutic strategies for peripheral nerve injuries in clinical contexts. The small sample size is one of its limitations. The small size was due to a lack of funding. And the second limitation of this study is that we only evaluated functional recovery without morphological or histochemical studies. Future studies could incorporate techniques such as magnetic resonance microscopy imaging, optical projection tomography, or high-resolution ultrasound [37].

## 5. Conclusions

The combination of PDRN and LLLT markedly improves facial nerve recovery after crush injury, as evidenced by enhanced vibrissa fibrillation, the recovery of action potential thresholds, and increased nerve blood flow. These results indicate that PDRN and LLLT may work synergistically to facilitate peripheral nerve regeneration. Future research should investigate the molecular mechanisms involved and evaluate the potential clinical applications of this combined treatment approach.

## Data Availability

The data presented in this study are available upon request from the corresponding author.
